# A Dynamic Overview of Antimicrobial Peptides and Their Complexes

**DOI:** 10.3390/molecules23082040

**Published:** 2018-08-15

**Authors:** Viviane Silva de Paula, Ana Paula Valente

**Affiliations:** 1 Department of Chemistry and Biochemistry, University of California Santa Cruz, Santa Cruz, CA 95064, USA; vsilvade@ucsc.edu; 2 Centro de Biologia Estrutural e Bioimagem, Instituto de Bioquímica Médica, Centro Nacional de Ressonância Magnética Nuclear Jiri Jonas, Universidade Federal do Rio de Janeiro, Rio de Janeiro 21941-902, Brazil

**Keywords:** antimicrobial peptides, defensins, NMR, structure and dynamics, conformational selection

## Abstract

In this narrative review, we comprehensively review the available information about the recognition, structure, and dynamics of antimicrobial peptides (AMPs). Their complex behaviors occur across a wide range of time scales and have been challenging to portray. Recent advances in nuclear magnetic resonance and molecular dynamics simulations have revealed the importance of the molecular plasticity of AMPs and their abilities to recognize targets. We also highlight experimental data obtained using nuclear magnetic resonance methodologies, showing that conformational selection is a major mechanism of target interaction in AMP families.

## 1. Introduction

The biological functions of proteins are coded in their structures, and dynamic and conformational changes are key events in all processes that involve protein recognition [1,2,3,4]. Nuclear magnetic resonance (NMR) and molecular simulations have shown that protein structure is heterogeneous and can adopt various conformational states that are energetically similar, separated by energy barriers on the order of kT (the product of the Boltzmann constant, k, and the temperature, T) [2,3,5,6]. In the presence of a ligand, a specific conformation is trapped, characterizing a conformational selection mechanism [7,8]. In the unbound state, a protein coexists with the bound state in some small fraction, and this fraction can bind to the ligand to reach the fully bound state. Further accommodation can occur, characterizing an induced-fit event [9]. To allow crossing at physiological temperatures, the barriers must be small because the dynamics are driven by the thermal fluctuations of the surrounding liquid [2,5]. Additionally, loss of entropy in the protein, which opposes binding, is often paired with disorder in remote regions, usually not correlated with the binding site in addition to changes in entropy of the solvent. New flexible regions often arise in the relaxed bound state or protecting exposed hydrophobic region and increasing the entropy in the well-known enthalpy-entropy compensation mechanism [2,10]. Regions with multiple states in the conformational space are accessed via local thermal fluctuations, and they play important roles in the entropic-enthalpic balance during recognition and binding. By identifying and analyzing the regions of local flexibility and cooperative motion of the residues inside a protein, we argue that it is possible to determine which parts of the protein will lead the kinetics of biologically relevant processes. These regions are typically characterized by highly conserved residues within a family of proteins with related biological functions and are involved in binding interactions [2,3,10].

This complex behavior occurs across a wide range of time scales and has been challenging to portray. These changes can range from side chain motions to domain reorientation; they therefore occur at differing time scales and energy barriers. Recent advances in NMR and molecular dynamics have provided experimental evidence and offer direct measurement of protein motion in solution at atomic resolutions [11].

In this review, we focus primarily on applying NMR methods to the elucidation of interactions between antimicrobial peptides (AMPs) and their targets. We summarize the methods that can be used to reveal molecular mechanisms of interaction, beginning with structure determination using NMR and proceeding to a high-resolution view of the structures and dynamics of their complexes.

## 2. Antimicrobial Peptides

All multicellular organisms produce AMPs as a first line of defense. More than 2500 AMPs have been deposited in the Antimicrobial Peptide Database (http://aps.unmc.edu/AP/main.php), and they represent a diversity of activities against bacteria, yeasts, fungi, viruses, and cancer cells [12,13]. Although their mechanisms of action are not completely understood, a large number of AMPs act with high selectivity and specificity through interactions with membrane lipid components and components of the cellular matrix, targeting cytoplasmic components and interfering with cellular metabolism [12,13,14]. In solution, these peptides undergo complex conformational changes related to their multiple targets and mechanisms of action [15,16,17]. This review is mainly about defensins, which are AMPs, but not all AMPs are proteins (but rather short peptides) and not all AMPs adopt a well-defined structure, unless in the presence of a membrane.

Defensins represent a class of cationic AMPs that play pivotal roles in innate and adaptive immunities against microbial and viral infections and play various roles in inflammation, wound repair, expression of cytokines and chemokines, production of histamine, and enhancement of antibody responses [17]. They constitute an ancient and diverse gene family, present in most multicellular organisms ranging from plants, fungi, insects, mollusks, and arachnids to mammals, including humans.

Defensins are a family of small, conserved proteins (~5 kDa). They are cysteine-rich AMPs and are classified into three subgroups, α-, β-, and θ-defensins, based on their amino acid sequences and patterns of disulfide bond connectivity [18,19,20,21,22]. All defensin structures contain a β-sheet fold that is stabilized by three disulfide bonds formed by six cysteine residues [23]. β-Defensins contain three intramolecular disulfide bonds in a I–V, II–IV, III–VI arrangement [24,25]. Although genome-based analyses have identified 28 human genes codified for human β-defensins (HBDs) [26], to date, only a few have been isolated and studied at the protein level. Human β-defensins 1–4 are predominantly secreted by epithelial cells and the male reproductive tract [27]. Both HBD5 and HBD6 appear to be specifically expressed in the epididymis [28].

**Figure 1:**
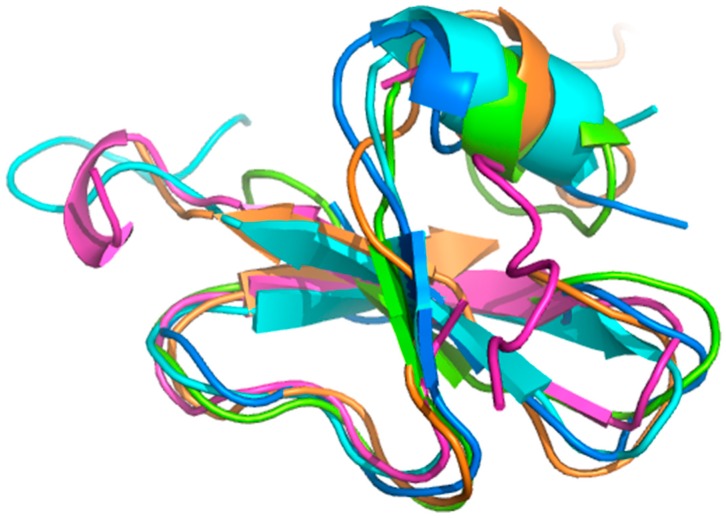
Superposition of reported structures for five human β-defensins (HBDs). Represented are HBD1 (protein data bank 1KJ5, blue), HBD2 (1FQQ, green), HBD3 (1KJ5, orange), HBD4 (5KI9, pink), and HBD6 (2LWL, cyan). Structure alignment was generated using the software jFATCAT [29].

Even though sequence identity between defensins varies from about 20% to 90%, and significant differences are evident in their biological functions, all defensins described thus far adopt essentially the same fold. Figure 1 shows a superposition of five defensins (HBD1, HBD2, HBD3, HBD4, and HBD6) to illustrate this similarity. Each of these structures consists of an N-terminal α helix and a three-stranded antiparallel β sheet. The root mean square deviation (RMSD) value across the Cα backbone atoms for these five β-defensin structures is 1.99 Å, but this varies within the N-terminal region due in part to increased flexibility (see Section 2.1).

The highly conserved cysteine residues pair up to form disulfide bridges that are crucial to maintaining structural integrity, which is a prerequisite for defensin activity.

### 2.1. Conformational Dynamics of Defensins

Nuclear magnetic resonance is currently the most powerful technique used to characterize the structural and dynamic properties of AMPs in complexes with their potential molecular targets, such as micelles and other membrane mimetics or oligosaccharides and peptides. To apply advanced NMR techniques, relatively large amounts of isotope-labeled proteins containing ^13^C, ^15^N, or ^2^H are desired [30,31]. Therefore, a number of methods for producing recombinant AMPs in bacteria have been developed [32,33,34]. Recombinant AMPs can be challenging to express because they can be toxic to their host bacteria and susceptible to proteases. We successfully expressed five β-defensins (HBD1, HBD4, HBD5, HBD6, and HBD11) as soluble fusion proteins using thioredoxin in *Escherichia coli* [32] (for HBD1 and HBD6). The pET32 vector uses thioredoxin as a fusion partner, increasing expression level, stability, and solubility [35].

Recent NMR studies of defensin dynamics [32,36,37] have revealed that these heavily cross-linked small proteins exhibit extensive conformational dynamics. Micro- to millisecond conformational exchanges are extensive, attributable to transient interconversions between the native ground state and energetically less-favorable ‘excited’ state(s). Studies have shown that transitions to such sparsely populated conformers are implicated in many aspects of protein function [38,39,40].

**Figure 2:**
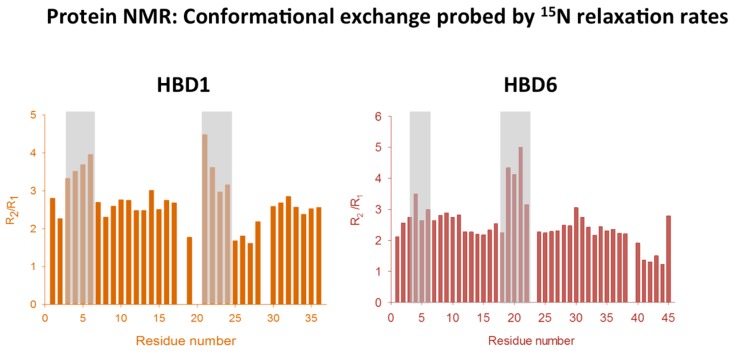
Backbone dynamics of β-defensins. Analysis of ^15^N spin relaxation parameters revealed regions undergoing microsecond to millisecond motions. Note grey areas for a direct comparison between regions of conformational exchange in the two β-defensins. Figure adapted from [32,41].

Interestingly, HBD1 and HBD6 ^15^N backbone relaxation analyses (Figure 2) identified regions undergoing microsecond to millisecond motions encompassing the α-helix and the loop between β1 and β2 strands [32]. These data showed that the conformational exchanges undergone by HBD1 and HBD6 could represent a mechanism conserved across the β-defensin family. This study emphasizes the conformational plasticity in β-defensins, which can play an important role in receptor interactions and is an important consideration in engineering defensins. However, only two β-defensin dynamics have been characterized thus far, circumventing extrapolation to other β-defensins.

### 2.2. Sugar Cane Defensin 5

**Figure 3:**
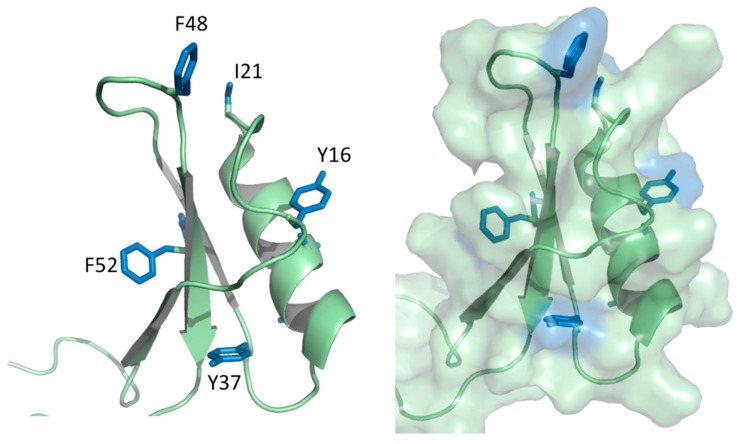
Hydrophobic residues arising from the anti-parallel β-sheet and α-helix of sugar cane defensin (SD)5. Close-up views of the SD5 structure (cartoon and surface representations) showing side chains of the hydrophobic residues. Note that all hydrophobic residues are surface exposed. The aromatic residues located at the secondary structure elements are indicated.

This complex behavior has also been observed in sugar cane defensin 5 (SD5), which exhibits complex millisecond conformational dynamics involving all secondary structure elements [37]. Temperature-dependent Carr-Purcell-Meiboom-Gill NMR relaxation dispersion measurements probed the sparsely populated “excited” state of SD5, revealing it to be enthalpically unfavorable, suggesting a rearrangement of stabilizing contacts formed by surface-exposed side chains and/or a secondary structure. Overall, the emerging depiction of SD5 dynamics suggests this protein can populate either of two well-ordered conformational states, the excited conformer being more compact than the native state, and having a distinct secondary structure and side chain arrangements. The observation of an energetically unfavorable yet more compact excited state reveals a remarkable evolution of the CS α/β fold to expose and reorganize hydrophobic residues (Figure 3), enabling the creation of versatile binding sites and mechanisms for enthalpic-entropic compensation [41,42].

### 2.3. Dynamics Obtained Using Molecular Dynamics Simulations

Few defensin dynamics are characterized using NMR parameters; however, interesting results have been obtained using molecular dynamics. Sharadadevi and Nagaraj studied HBD1 and HNP-3 in water, and they observed structural fluctuations even in the presence of disulfide bonds [43]. Furthermore, both these entities unfold during MD. One interesting structural feature of HBD1, according to NMR data, is the lack of strong and well-defined interactions between secondary structure elements [44]. Also, deuterium exchange does not indicate the presence of extensive hydrogen bonding between the β-strands [45]. Also, common structural feature among defensins is their lack of hydrophobic cores and lack of extensive hydrogen bonding, implying conformation plasticity, an important characteristic for biological functions involving differing targets. The structure is stabilized primarily by disulfide bonds, allowing it to tolerate extensive residue substitution without disturbing the final tertiary structure. Molecular dynamics simulations performed by Zhang et al. revealed microsecond-long motions in α-defensin 5 and β-defensin 3 [45]. As also shown by their NMR results, despite the presence of disulfide bonds, these defensins present dynamic properties. Toubar et al. observed sliding monomers in dimer formation among HBD1 and mutants, suggesting that in other forms of oligomers as well, dynamics are observed and found to be important for the biological functions of HBD1 [46].

### 2.4. Analyzing Defensin Complexes Using NMR

**Figure 4:**
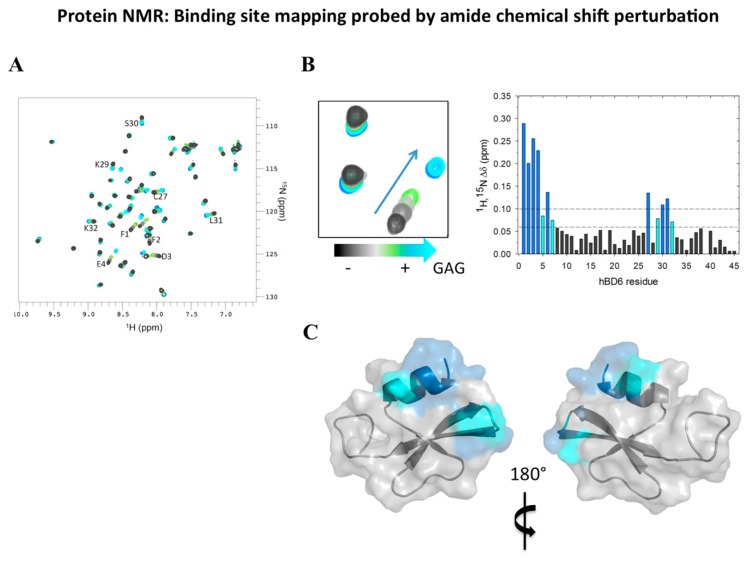
Nuclear magnetic resonance (NMR) as a tool to determine ligand-defensin complex formation. (**A**) Once the backbone chemical shift assignment is accomplished, a ^1^H-^15^N heteronuclear single quantum coherence (HSQC) spectrum of the free protein containing the assigned amide groups is obtained. (**B**) Titration of an unlabeled ligand (in this example, a glycosaminoglycan [GAG]) bound to ^15^N-labeled human β-defensin (HBD)6 results in a chemical shift perturbation map of HBD6-ligand interactions. The NMR titration revealed large changes in the chemical shift of HBD6 in a fast-exchange equilibrium between free and GAG-bound HBD6, as demonstrated by a gradual shift in peak positions in ^1^H-^15^N HSQC spectra. (**C**) The amide groups significantly perturbed upon GAG binding are highlighted on the HBD6 surface (protein data bank: 2LWL) in blue and cyan to map the binding sites for the GAG. Figure adapted from [41]. For further details see Section 2.5.

The primary advantage of NMR is its ability to detect and analyze dynamic systems. Biomolecular NMR applications demand a combination of experiments applied to isotope-labeled samples. To map a ligand-protein binding site within a protein, ^1^H-^15^N correlation experiments are typically used, and this requires that the protein be ^15^N labeled. These fingerprint spectra of the protein can be easily recorded, and they allow tracking of the ligand binding event at residue resolution (Figure 4). 

Once the backbone chemical shift assignment is accomplished, chemical shift analysis gives information about the structure [47,48]. These chemical shift perturbations (CSPs) are then mapped onto the protein structure to define binding sites—regions that were perturbed in the presence of the ligand (Figure 4C). Changes in peak line width and intensity are also useful, reflecting changes in size and/or dynamics.

Changes in the rotational correlation time (determined using NMR relaxation times) or the translational self-diffusion coefficient (measured using pulsed-field gradient NMR diffusion methods) have also been used to detect binding and dimerization and to assess the K_d_ value of the dimer. Analysis of NMR data offers structural insights into ligand-protein interactions at residue resolution. The information obtained is used to define areas of mutagenesis, guiding the design of functional studies, etc.

### 2.5. Defensin Interactions with Glycosaminoglycans and the Role of Dimerization

Glycosaminoglycans (GAGs) are sulfated polysaccharides, important components of the extracellular matrix. Several studies have established that cellular trafficking is orchestrated through chemokine-GAG interactions, which dictate the duration and steepness of chemokine gradients [49,50,51,52]. Although the roles of chemokine oligomerization and GAG binding have been known for some time, details of how defensins are recognized and immobilized by GAGs remain elusive. Of the five β-defensins for which 3-dimensional structures are currently known, only HBD2 and HBD6 have been characterized, via NMR, in their abilities to bind to GAGs [41,53].

**Figure 5:**
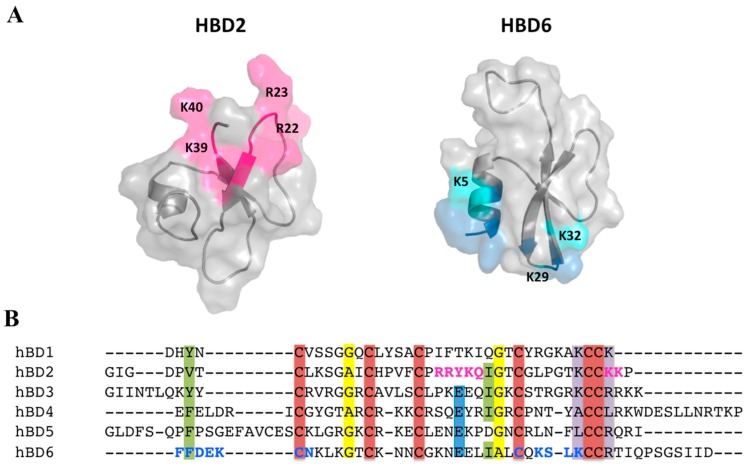
β-Defensin interaction with glycosaminoglycan (GAG), mapped using nuclear magnetic resonance (NMR) chemical shift perturbation (CSP). (**A**) Comparison of the heparin-binding motif mapped on the surfaces of human β-defensin (HBD)2 and HBD6. Residues exhibiting pronounced NMR CSP upon GAG binding are highlighted in magenta for HBD2 [53] and blue for HBD6 [41] on the surface and in the primary sequence. The CSPs for HBD2 were reproduced from [53]. (**B**) Sequence alignments for six HBDs, performed using ClustalW software. Protein names are indicated on the left; amino acids are color-coded based on conservation scores 9, calculated using the ConSurf server. Highly conserved Cys, Gly, Glu, and basic residues are shown in red, yellow, blue, and purple backgrounds, respectively.

For a range of GAGs, including heparin/heparan sulfate, dermatan sulfate, and chondroitin sulfate, Seo et al. elucidated the sites where binding on HBD2 takes place. Using NMR CSP analysis, five basic residues (Arg22, Arg23, Lys25, Lys39, and Lys40) of HBD2 were found to be involved in the formation of complexes [53]. In addition, HBD2 dimerization has been characterized using mass spectrometry and hydrodynamic calculations, establishing that it is induced by GAGs. Interestingly, the three basic residues in the ^22^RRYK^25^ stretch form a BBXB (B representing a basic amino acid, and X representing any amino acid) heparin-binding motif, which together with K39 and K40, creates a positive patch on the HBD2 surface (Figure 5A). This identified binding site is closely similar to the heparin binding site of the chemokine RANTES [54], which also contains the BBXB motif (^44^RKNR^47^) together with two additional spatially proximate basic residues, R20 and K25 [53].

In our studies of HBD6, we used a combination of structural and in silico analyses to characterize its interactions with GAGs [41]. We found that the HBD6-GAG complex reveals residues located at the α-helix and the loop between the β and β strands comprising the oligosaccharide binding interface. Interestingly, residues Lys-29, Ser-30, and Lys-32 showed increased R2 values in the presence of GAGs compared to free HBD6 in solution. Another interesting feature of this defensin is that the picosecond to nanosecond dynamics observed in the first residues located in the α-helix in the free state were reduced in the GAG-bound form, indicating that the α-helix becomes more structured upon GAG binding. These data suggest that Ser-30, Lys-5, Lys-29, and Lys-32, are parts of the BBXB motif, a known feature observed in heparin-binding proteins (Figure 4 and Figure 5A). Additionally, our data suggest a dynamical compensation in the structure upon complex formation. Finally, high-pressure NMR and relaxation parameters showed that GAG binding modulates multiple structural and dynamic properties of HBD6, inducing formation of a ternary complex with 2:1 HBD6:GAG stoichiometry (see Figure 5).

The low sequence identity (17%) between HBD2 and HBD6 imposes difficulties in identifying regions important for dimerization and GAG interaction; nevertheless, the same basic residues patch-mapped in HBD2 are conserved in HBD3 and HBD6 (Figure 5B). The basic patch is formed by various residue types and could explain differences in interactions.

### 2.6. Modeling Defensin Complexes Using High Ambiguity Driven Docking

Nuclear magnetic resonance allows in vitro structural studies of protein complexes [55]; however, elucidation of entire structures is challenging because they are large in size and dynamics at interfaces between subunits leads to exchange-induced line broadening in the spectra. Determining and quantifying binding and cooperativity in protein complexes is challenging and requires a combination of biophysical methods and integrated structural biology methodologies. Computational docking approaches using experimental data have been used to generate models of a complex based on known 3-dimensional structures of the components in their free states. The high ambiguity driven docking (HADDOCK) method makes use of CSPs to model the structures of protein complexes [43,56,57,58,59,60]. Specifically, differences in the backbone and side chain chemical shifts upon complex formation are used to derive ambiguous distance restraints [61,62], thus driving the subunits to dock, given the changes are localized to the binding surface.

**Figure 6:**
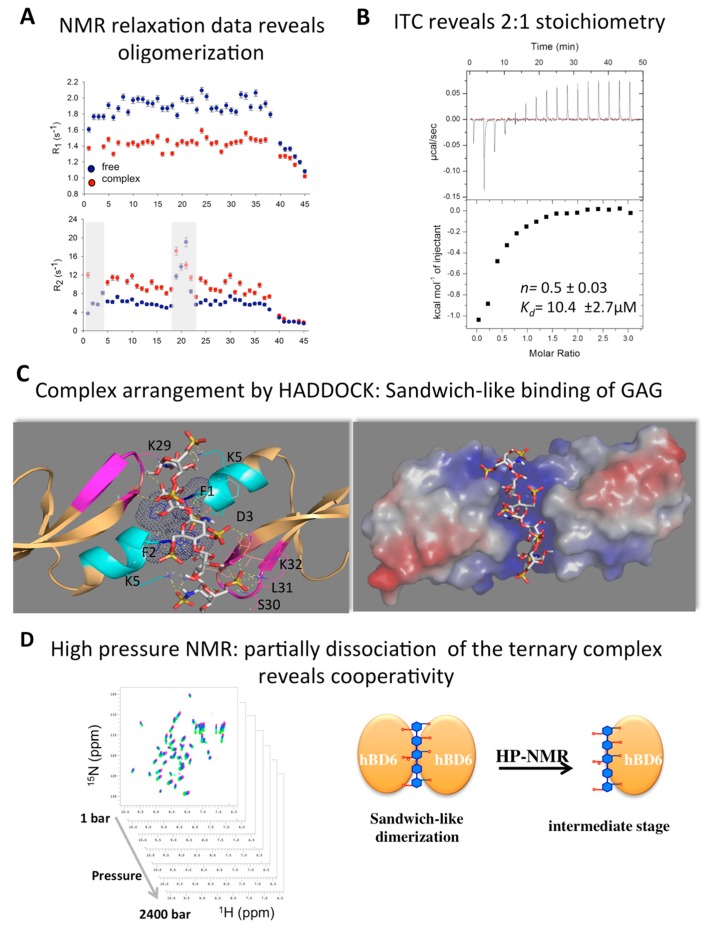
Dimerization and cooperativity during glycosaminoglycan (GAG) recognition by human β-defensin (HBD)6. (**A**) NMR relaxation experiments can reveal conformational exchanges and oligomerization (determined using NMR relaxation times) of defensins alone or complexed with GAGs. The R_2_/R_1_ ratios are translated during changes in the rotational correlation time, revealing the size of the complex. Note the grey areas for a direct comparison of the R_2_ values between the two HBD6 states (free and GAG-bound). (**B**) ITC measurements dissect the binding mechanism of HBD6 and confirm the 2:1 stoichiometry. (**C**) Rendition of the “sandwich-like” dimerization mechanism. The lowest-energy structure of the HBD6-GAG complex within the highest-scoring NMR-restrained high ambiguity driven docking alternatives is chosen. Residues with substantial chemical shift perturbations upon GAG titration are highlighted in cyan (α-helix) and magenta (β2-β3). Hydrogen bonds and salt bridges formed by the sulfate moieties are represented by yellow dashed lines. Hydrophobic interactions between the aromatic rings of F1 residues from each monomer of HBD6 are represented by dark blue dots (right panel). The electrostatic potential of the ternary complex is indicated. Surface polarity is indicated as positive (blue), negative (red), or nonpolar (white; left panel). (**D**) High-pressure NMR confirms the sandwich-like dimerization mechanism and the induced dissociation of the HBD6-GAG ternary complex, revealing an intermediate stage. A series of ^1^H-^15^N heteronuclear single quantum coherence spectra were recorded, varying the pressure. This data can reveal a snapshot of the first stage of GAG recognition whereby HBD6 exhibits features of a cooperative binding mechanism. This panel is adapted from [41] with permission from the original publisher.

The defensin system complexed with various ligands has been explored using a combination of structural biology methodologies and docking guided by NMR CSP data [32,41]. Using HADDOCK, a rendition was created, and it indicates that two HBD6 monomers sandwich the GAG. When combined with high-pressure NMR and ^15^N relaxation data, HADDOCK is a powerful tool, capturing intermediate stages that could represent snapshots of the first stages of recognition and ligand binding [41] (Figure 6).

The importance of both defensin-GAG interactions and defensin oligomerization remains poorly understood. To define the defensin-GAG functional role in innate and adaptive immune responses, additional structural and cellular studies are necessary.

Recently, Jarva et al. [63] showed that the dimerization interface of HBD2 is one of the binding sites of the lipid phosphatidylinositol 4,5-biphosphate (PIP2). The X-ray structure of HBD2 in the presence of PIP2 revealed two binding sites and shed important information in the mechanism of action of defensins.

### 2.7. Challenges to Understanding AMPs and Membrane Interaction

Antimicrobial peptides are usually short and cationic and exhibit extensive conformational diversity. Structure determination is often problematic because of their intricate dynamics, resulting in calculated structures with low convergence and geometrical violations [12,64,65,66]. On the other hand, characterization of AMP structures and dynamics is important so that their mechanisms of action can be understood, leading to the strategic design of new pharmacologically active compounds [15,16,67].

Membrane interaction is the first event in the AMP mechanism of action, and it is accompanied by a decrease in dynamics and acquisition of an amphipathic conformation. The AMP mechanism of action has been modeled by the barrel-stave model, the carpet model, and the toroidal pore model, among others. Solid-state NMR makes important contributions to the understanding of AMP organization and disruption in membranes [68,69,70,71,72]. One of the most-studied AMPs, magainin, lies in the bilayer interface and loosens the packing of the hydrophobic region, reducing the bilayer thickness [67]. Various studies confirm that the order parameter of the bilayer core decreases using deuterium NMR, possibly suggesting the most probable mechanism of disruption, but the complete picture is not yet known. Mani et al. used ^19^F to show lipid disorder near the toroidal pore formed by protegrin-1 [69].

Paramagnetic Relaxation Enhancement (PRE) measurements can also provide information about AMPs inserted in micelles and membranes [73]. Taken together, these parameters can aid in the interpretation of the relationships between PRE values and peptide orientations in micelles.

To contribute to this work, we examined tritrpticin (TRP3), a cathelicidin AMP that exists as an ensemble of four conformations in solution [74]. The indole region therefore produces 12 peaks in ^1^H NMR spectra rather than the expected three. In the presence of micelles, one major conformation is stable [74,75,76,77].

Using NMR to evaluate the structure of TRP3 in micelles with various chemical features reveals interactions occurred through conformational selection, stabilizing similar structures despite differences in detergent headgroups or dipole orientations [78]. These data also reveal the imperative constraint of the WWW-F motif in the TRP3 structure, acquired at interfaces, and show that pre-organization in the free state is crucial for membrane interaction.

A large number AMPs act with high selectivity and specificity through interactions with membrane lipid components. These peptides undergo complex conformational changes in solution, but upon binding to an interface, one major conformation is stabilized.

A number of theoretical and computer simulation approaches have been established to describe AMP and lipid interactions. The MD is an “in silico” approach that can characterize the interaction between AMPs and membranes, in which the dynamics of the molecule into the lipid bilayer and its affects on surrounding lipids are explored. MD simulations have succeeded in precisely describing the process of peptide binding, folding and partitioning into lipid bilayers, at atomic resolution [79,80].

Recently, Wang et al. (2015) used experimentally guided unbiased MD in order to characterize the molecular mechanisms of pore formation by maculatin, a well-known AMP from the Australian tree frog *Litoria genimaculata*, revealing that rather than a single pore or detergent-like holes, maculatin forms an ensemble of structurally different channels that continually form and disperse at equilibrium [81,82]. Membrane permeabilization is dominated by oligomeric forms of maculatin, which conduct water, ions and small dyes.

Lee et al. (2016) explored the adsorption mechanism of human β-defensin 3 (HBD3) using Gram-negative (GN), Gram-positive (GP), and mammalian membrane models, through MD simulations [83]. They reach to the conclusion that Arg17, Arg36 and Arg38 form both polar and nonpolar interactions and are potentially the key residues for HBD3 antibacterial activity. Also, they concluded that high concentration of POPG lipids in the GP membrane leads to strong electrostatic interaction with HBD3 and stabilize its adsorption rapidly as compared with HBD3-GN membrane complex.

## 3. Conclusions

Antimicrobial peptides are important for innate and adaptive immune responses, and they can provide a variety of avenues in drug discovery. The universe of biological functions is linked to the binding promiscuity of AMPs, which is related to their ability to oligomerize and to interact with membranes and elements of the cellular matrix. All possibilities are correlated to their dynamical properties because regions that have multiple states are associated with recognition and binding. By identifying and analyzing the regions of local flexibility and cooperative motion of the residues inside a protein, those parts of the protein that lead the kinetics of the biologically relevant processes can be elucidated.
